# Outcome of COVID-19 in Egyptian living-donor kidney transplant recipients and relation to maintenance immunosuppressive drugs: a pilot study

**DOI:** 10.1038/s41598-023-45750-8

**Published:** 2023-11-03

**Authors:** Maggie Said ElNahid, Marianne Samir Makboul Issac, Khaled Marzouk Sadek

**Affiliations:** 1 Department of Internal Medicine and Nephrology, Faculty of Medicine, Cairo University, Cairo, Egypt; 2 Department of Clinical and Chemical Pathology, Faculty of Medicine, Cairo University, Cairo, Egypt

**Keywords:** Medical research, Nephrology

## Abstract

Coronavirus disease 2019 (COVID-19) in kidney transplant recipients is a subject of much debate and became of interest to nephrologists amidst the pandemic. The main concerns are the influence of the chronic use of immunosuppressive drugs, the viral-related risk of acute rejection, and the long-term outcome of allograft function. This single-center prospective study included kidney transplant recipients with COVID-19 infection. Patients were maintained on immunosuppressive regimens. The severity of disease was defined as oxygen saturation < 94%, the need for hospitalization and/or hemodialysis, the occurrence of acute kidney injury (AKI), and mortality. Seventeen patients (54.8%) required hospital admission, four patients needed hemodialysis (12.9%), twelve patients (38.7%) had AKI, and three patients died (9.7%). Oxygen saturation < 94% showed a positive correlation with the presence of diabetes (p value 0.031) and a negative correlation with the maintenance steroid dose (*p* value 0.046). A negative correlation existed between the need for hemodialysis and average Cyclosporin level (*p* value 0.019) and between the need for hospitalization and average Tacrolimus level (*p* value 0.046). Severity of disease was associated with the presence of lymphopenia (*p* value 0.042), the cumulative steroid dose (*p* value 0.001), increased serum levels of LDH (*p* value 0.010), Ferritin (*p* value 0.020), AST (*p* value 0.047), and ALT (*p* value 0.006) and D-dimer levels more than 0.5 mg/L (*p* value 0.038). This study highlighted that the immunocompromised state of renal transplant recipients may not be regarded as a disadvantage in the setting of COVID-19 infection. Studies on a larger scale are needed to validate these results.

## Introduction

It is undetermined whether COVID-19 infection in the transplant population has similar symptoms and outcomes compared to the immunocompetent population^1^. The study of the transplant population is warranted to investigate the impact of maintenance immunosuppressive therapy on COVID-19 infection^2^. Unfortunately, data on allograft function in kidney transplant recipients with COVID-19 infection is still limited^3^. Since in vitro studies of Calcineurin inhibitors with SARS-COV yielded promising results, it is essential to investigate COVID-19 outcomes in similar settings^4^. The present study aims to describe COVID-19 infection among Egyptian living-donor kidney transplant recipients and to highlight the impact of long-term immunosuppression on the severity and outcome of the infection.

## Methods

This prospective study included living-donor kidney transplant recipients who tested positive for COVID‐19, from a single center in Cairo, Egypt, between March 20, 2020, and March 20, 2021. All procedures followed are per the Helsinki Declaration of 1964^5^. This study was approved by the Department of Internal Medicine local ethical committee at Cairo University, Egypt.

### Data collection

Demographic data (age, sex, BMI, comorbidities, duration of post-transplant follow-up and maintenance immunosuppression drug levels), clinical characteristics (presenting symptoms, examination findings, laboratory results, and chest C.T. results), medications during COVID-19 (antiviral, antibiotic, anticoagulation treatment, and oxygen therapy), complications (Acute Kidney Injury (AKI), hospitalization, hemodialysis and Intensive care unit (ICU) admission) and outcome (recovery or death) were recorded for all participants. Information about hospitalized patients was gathered by the inpatient team. Baseline kidney function was considered as the mean of the patient's last three serum creatinine results before COVID-19 infection. AKI was defined according to the Kidney Disease Improving Global Outcomes (KDIGO) guidelines^6^. Cyclosporine (CsA) drug level measurement was done 2 h post-dose (C2)^7^.

### COVID‐19 diagnosis

Diagnosis of COVID-19 infection was based on clinical, laboratory, and radiological findings. Confirmation of diagnosis was made by real-time polymerase chain reaction assay of nasopharyngeal swabs and the complementing findings on a chest C.T. scan^8,9^. Laboratory investigations included a complete blood count, serum C-reactive protein, LDH, ferritin, basal INR, kidney and liver function tests, and electrolytes, D-dimer, CsA, or Tacrolimus (Tac) drug level.

### Patients

Informed consent was obtained from all participants. Patients were diagnosed according to the severity of the disease as per the protocol issued by the Egyptian Ministry of Health and Population guidelines at the time^10^. Patients with mild disease, who have an oxygen saturation of more than 94% and no lung involvement on chest C.T., were followed up on an outpatient basis. Corticosteroid therapy was not prescribed for these patients. Indications for hospitalization were moderate to severe disease, characterized by oxygen saturation less than 94%, respiratory rate more than 30 /min, positive chest findings on C.T., or the need for hemodialysis. Cytokine storm was diagnosed by persistent fever, high CRP and ferritin levels, abnormal liver function tests, D-dimer >0.5mg/dL, lymphopenia, and thrombocytopenia. ICU admission was based on low oxygen saturation of less than 90%, Systolic Blood Pressure of less than 90 mm Hg, or dysfunction in multiple organs.

Early in the pandemic, the most commonly used drugs were hydroxychloroquine, protease inhibitors, and Azithromycin^11^. Later, only Remdesivir became approved for the treatment of COVID-19^12^. A contraindication to its use was an estimated glomerular filtration rate (eGFR) of less than 30 mL/min/1.73 m^2^. Hydroxychloroquine was administered orally at a dose of 400 mg twice a day for the first 2 days and then 200 mg twice a day for four days, with routine ECG monitoring. Azithromycin was administered orally at a dose of 500 mg once a day for the first 3 days, then 250 mg once daily for four days. The FDA warned of a threefold increase in CsA level with concomitant use of chloroquine^13–15^ and advised for dose reduction in the presence of renal impairment, with a maximum of 5 days of treatment. Chloroquine is also known to cause a prolonged QTc-interval. Therefore, ECG monitoring was done regularly to these patients, especially in cases of renal impairment^16^.

Steroids were administered to patients with an oxygen saturation < 94%. Patients with moderate or severe disease received Tocilizumab or Remdesivir treatment and were treated at isolation hospitals. The corticosteroids were administered as dexamethasone 6 mg or the oral equivalent in moderate cases and methylprednisolone 1 mg/kg/d together with Tocilizumab 4–8 mg/kg/d in 2 doses, 12 h apart, and Remedesivir 200 mg as a 1st dose then 100 mg/d for 5 days in severe cases^17–21^. Drug interaction with immunosuppressive drugs was revised. Antibiotic therapy was added in cases of bacterial infection^22,23^.

A D-dimer level of more than 0.5 mg/L was considered an indication of anticoagulation. Prophylactic-dose low-molecular-weight heparin or Enoxaparin 40 IU every 24 h were administered subcutaneously to critically ill patients. Renal dose adjustments were done when needed. On an outpatient basis, anticoagulation was administered as Apixaban 2.5 mg PO bid or Rivaroxaban 10 mg daily for 6 weeks^24^. The immunosuppressive regimen was restored after a month of infection after recovery.

### Statistical analysis

Data were statistically described in terms of mean ± standard deviation (± SD), median and interquartile range, or frequencies (number of cases) and percentages when appropriate. A comparison of numerical variables between the study groups was done using the Mann–Whitney *U* test for independent samples. For comparing categorical data, Chi-square (χ^2^) test was performed. An exact test was used instead when the expected frequency is less than 5. The Correlation between variables was done using the Spearman rank correlation equation for non-normal variables/non-linear monotonic relation. A two-sided *p* value less than 0.05 was considered statistically significant. All statistical calculations were done using the computer program IBM SPSS (Statistical Package for the Social Science; IBM Corp, Armonk, NY, USA) release 22 for Microsoft Windows.

## Results

### Patient baseline characteristics

From March 2020 to March 2021, among kidney transplant recipients, 31 had confirmed COVID-19 infection, 18 males (58.1%) and 13 females (41.9%) with an age range of 30–78 years old and a range of 2–18 years duration after kidney transplantation. All had received a living-donor kidney transplantation. None of the patients received induction therapy.

Eight patients had diabetes (25.8%), 19 had hypertension (61.3%), and five patients had ischemic heart disease (16%). Patients were maintained on immunosuppressive regimens, 17 patients were on CsA (54.8%), 14 patients on Tac (45.2%), two patients on Azathioprine (6.4%), and 29 on Mycophenolate (93.5%). The average Tac level was 6.04 ± 1.083 ng/dL, while the average CsA level (C2) was 424.06 ± 89.437 ng/dL. The median serum creatinine was 1.65 (1.09–1.5). Demographic, laboratory characteristics and immunosuppression regimen of the participants are shown in Table 1.

**Table 1:** Baseline characteristics of kidney transplant recipients with COVID-19.

	N	Minimum	Maximum	Mean ± S.D. or Median (25th–75th percentile)
Age (years)	31	30	78	51.23 ± 12.83
BMI (kg/m^2^)	31	22	36	27.74 ± 3.56
No. of years of Tx (years)	31	2	18	7.66 ± 4.13
CsA dose/day (mg/d)	17	25	200	114.71 ± 45.12
Tac dose/day (mg/d)	14	1	5	2.54 ± 1.12
AZA dose/day (mg/d)	2	100	150	125 ± 35.36
Steroid dose before COVID-19 (mg/d)	31	5	10	5.32 ± 1.07
Steroid dose during COVID-19 (mg/d)	20	40	200	160 ± 71.08
CsA Level (ng/dL)	17	269	580	424.06 ± 89.44
Tac Level (ng/dL)	14	4	8	6.04 ± 1.08
Baseline Creatinine (mg/dL)	31	0.9	5.9	1.65 (1.09–1.5)
Follow-up creatinine after COVID-19 (mg/dL)	31	1.0	9.0	2.30 (1.1–1.9)
LDH (U/L)	31	22	990	489.52 (200–693)
CRP (mg/L)	31	4	192	87.79 (70–102)
Ferritin (ng/mL)	31	18	1230	646.29 ± 433.55
ALT (U/L)	31	17	200	45.00 (30–50)
AST (U/L)	31	12	150	41.16 (30–43)

Data are presented as mean ± SD or median (interquartile range).

### Clinical presentation

During the COVID-19 infection, all patients had fever (100%), 24 had non-productive cough (77.4%), 15 had gastrointestinal symptoms (48.4%), 17 patients suffered a severe attack, with an oxygen saturation < 94% (54.8%), 26 patients had lymphopenia evident in the complete blood count (83.9%), 22 had a D-dimer > 0.5 mg/L (71%) and 29 (93.6%) of cases had a high CRP. Twelve patients were diagnosed with AKI (38.7%), 17 patients were hospitalized (54.8%), and four patients needed hemodialysis (12.9%).

### Treatment

The general approach to immunosuppressive therapy was mycophenolate withdrawal only^25^.

Regarding the treatment regimen, 27 patients were treated with hydroxychloroquine (87.1%), 22 received anticoagulation (71%), 3 patients received antiviral treatment, Remdesivir (9.7%). Renal dose adjustment was not necessary since the eGFR of all recipients exceeded 30 mL/minute. No adverse drug effects were recorded. Drug interaction with the immunosuppressive drugs was revised, and no interaction was recorded. Those who were eligible for Remdesivir treatment were not receiving hydroxychloroquine concomitantly. One patient received Tocilizumab 4–8 mg/kg/d in 2 doses 12 h apart. Regarding anticoagulation, patients already on anticoagulant or antiplatelet therapies continued these medications. Patients with deep venous thrombosis were treated with therapeutic-dose anticoagulation. Patients treated on an outpatient basis received Rivaroxaban 10 mg daily. Inpatients were treated with Enoxaparin 40 IU subcutaneously every 24 for prophylaxis. Patients whose oxygen saturation was less than 92%, were given supplemental oxygen.

### Outcomes

Regarding the outcome, 28 patients recovered (90.3%), while three patients died (9.7%). Improvement was confirmed on a clinical basis, normal follow-up C.T. and a twice-negative PCR. The median (IQR) follow-up (FU) creatinine after COVID-19 was 2.30 (1.1–1.9), Table 1.

### Association and correlation studies with severity

Correlation studies revealed a statistically significant positive correlation between oxygen saturation < 94% and the presence of diabetes (*p* value 0.031) and a statistically significant negative correlation with maintenance steroid dose (*p* value 0.046). There was a statistically significant negative correlation between the need for hemodialysis and the average CsA level (*p* value 0.019). A statistically significant negative correlation existed between the need for hospitalization and the average Tac level (*p* value 0.046).

In our study, oxygen saturation < 94%, the need for hospitalization, the development of AKI, and the need for hemodialysis were considered markers of severity. Patients were further stratified into two groups; 20 patients with severe disease and 11 patients without severe disease, based on the presence of any of these criteria as shown in Table 2.

**Table 2:** Association between the patients’ data and severity.

Patients’ data	Severity		*p* value
	No ( n=11)	Yes (n=20)	
Cumulative steroid dose (mg)	5 (5-205)	205 (5-210)	0.001
Baseline creatinine (mg/dL)	1.12 (1-1.2)	1.4 (0.9-5.9)	0.007
LDH (U/L)	276 (22-850)	605 (100-990)	0.010
Ferritin (ng/mL)	130 (18-1080)	960 (20-1230)	0.020
Lymphopenia	7 (26.9%)	19 (73.1%)	0.042
D-dimer > 0.5 mg/L	5 (22.7%)	17 (77.3%)	0.038
ALT (U/L)	30 (17-50)	40 (17-200)	0.006
AST (U/L)	32 (12-43)	39 (16-150)	0.047

Data are presented as frequencies (percentages) or median (range).

*p* value less than 0.05 was considered statistically significant.

Severity was associated with baseline creatinine (p value 0.007), the presence of lymphopenia (*p* value 0.042), the cumulative steroid dose (*p* value 0.001), increased serum levels of LDH (*p* value 0.010), Ferritin (*p* value 0.020), AST (*p* value 0.047), and ALT (*p* value 0.006) and D-dimer levels more than 0.5 mg/L (*p* value 0.038). Severity was not related to the number of years of transplantation.

## Discussion

As stated by the Centers for Disease Control and Prevention (CDC), chronic immunosuppression, the presence of comorbidities, and repeated contact with the healthcare system predispose kidney transplant recipients to severe COVID-19 disease^26^, although they may be more easily diagnosed during regular follow-ups at the transplant centers^27^. Only a few studies examined the COVID-19 infection pattern in kidney transplant recipients, and ideal immunosuppression remains to be determined^3^.

The present study aims to describe the COVID-19 infection among kidney transplant recipients and examine its impact on kidney function, the development of AKI, and the relationship of maintenance immunosuppressive drugs to the outcome and severity of COVID-19 infection. Our study was conducted on 31 living-donor kidney transplant recipients; 25.8% had diabetes, 61.3% had hypertension, and 16% had ischemic heart disease, similar to the known characteristics of transplanted patients^28^.

Previous studies stated that symptoms of COVID-19 infection in this population may be somewhat different^15,29^. Fever, dry cough, and fatigue are the most common complaints. Some patients may also experience dyspnea, muscle pains, sore throat, and GIT symptoms^30^. In some cases, abdominal pain occurs before respiratory symptoms^31^ or atypical symptoms without respiratory symptoms^32^. A few other studies reported that symptoms in transplant recipients do not differ from immunocompetent cases^33,34^. In a study by Ng et al., 55% to > 80% were hospitalized^35^.

In our study, all patients complained of fever (100%), 24 had a non-productive cough (77.4%), 15 had gastrointestinal symptoms (48.4%), and two (3.2%) experienced DVT. Seventeen patients suffered a severe attack, with an oxygen saturation < 94% (54.8%), 26 patients had lymphopenia, evident in the complete blood count (83.9%), 22 had a D-Dimer > 0.5 mg/L (71%), and 29 (93.6%) had a high CRP. Regarding the outcome, 12 patients had AKI (38.7%), 17 patients were hospitalized (54.8%), four patients needed hemodialysis (12.9%), 28 patients recovered (90.3%), and three patients died (9.7%).

COVID-19 can affect the kidneys in many ways; AKI is one of the most common forms^36^, which has a direct effect on mortality^37^. The incidence of AKI was reported as high as 85% ^38^. AKI was reported as 39.9% with 6.6% requiring dialysis, in a large study of almost 9000 patients. In a study by Jewell et al., renal replacement therapy (RRT) was 29.3% in transplant patients versus 5.8% in the general population^39^. The main risk factors for AKI include old age, diabetes, the severity of respiratory symptoms, the use of mechanical ventilation, and pre-existing renal disease^40^. The outcome of AKI in COVID-19 infection is related to the balance between the viral infection and the immunosuppressive dose adjustment^41^. In our study, the development of AKI was not related to any of the studied parameters; age, gender, BMI, diabetes, hypertension, IHD, or immunosuppressive drug levels.

In renal transplant recipients, mortality was recorded as 18% and 43%, higher than in the general population^42^. Risk factors associated with mortality included advanced age, high viral load, and high inflammatory markers. None of the parameters related to immunosuppression had an association with mortality^43^. Some studies, on the contrary, revealed better survival with the use of cyclosporine^44,45^. In our study, mortality was not related to age, gender, BMI, diabetes, hypertension, IHD, or immunosuppressive drug levels.

In our study, the observation of increased levels of inflammatory markers such as ferritin and LDH among the patients who developed severe disease suggests a direct relationship between the magnitude of cytokine-release characteristics of COVID-19 and the risk and severity of disease in renal transplant recipients. The association between high D-dimer levels or the requirement for anticoagulant administration with disease severity aligns with the prothrombotic state observed in COVID-19^46^.

Disease severity was associated with lymphopenia which is in accordance with previous observations^47^.

The observed elevation of transaminases in the severe-disease group is in agreement with previous reports showing evidence of a correlation between the severity of COVID-19 infection and liver enzyme elevation. Liver cells express ACE2 receptors where SARS-CoV-2 can directly bind to ACE2-positive cells and disrupt liver functions. Other causes might be drug-induced or hypoxic liver injury and systemic inflammatory response^48^.

There is a statistically significant positive correlation between oxygen saturation < 94% and the presence of diabetes (*p* value 0.031). This is in agreement with many previous studies. Wu et al. stated that patients with diabetes, infected with COVID-19, are at a much higher risk for ICU admission and mortality. Mortality among diabetic patients with COVID-19 (7.3%) was higher in comparison with nondiabetic subjects (2.3%)^49–51^. Severity and mortality were associated with the glycemic ratio^52–56^. Lim et al. suggested that hyperglycemia modulates β-cell dysfunction and promotes endothelial dysfunction and increases the formation of advanced glycation end products^57^. It causes exaggerated inflammatory responses, decreased antiviral activity, and decreased T-cell activation^50^.

In our study, results showed a statistically significant negative correlation between oxygen saturation < 94% and maintenance steroid dose (*p* value 0.046). Some studies reported that the use of steroids is beneficial to patients with COVID-19^58–63^. Others showed that steroid use was associated with a higher risk of bloodstream infections and a lower recovery incidence^64,65^ and a delay in viral clearance^66–71^. Kidney transplant recipients are mostly maintained on maintenance steroid therapy, rendering them vulnerable during this pandemic^72–74^. Nevertheless, many transplant experts keep the steroid dose during COVID-19 infection, a practice that is supported by many studies including the: RECOVERY study, Metcovid, and CoDEX trials^17,75–77^. Administration of steroids was linked to fewer mortality rates in some studies^75^ and did not affect outcomes in other studies^78^.

Most studies, however, examined the role of therapeutic steroids in the treatment of COVID-19 in terms of dose, timing of administration, and type of steroid used. To our knowledge, this is one of the very few studies that evaluated the relationship between *the maintenance* of immunosuppressive drugs and the severity of COVID-19 infection.

A study by Hadi et al. stated that the spectrum of COVID-19 in kidney transplant recipients is related to the type of induction and immunosuppressive treatment^72^. Other studies argued against any role played by maintenance immunosuppressive drugs^76^. Corticosteroids control the intense immune response which is responsible for lung damage in COVID-19 patients^17^, by inhibiting pro-inflammatory cytokines, stimulation of apoptosis of T-lymphocytes, and reduction of leucocyte recruitment^4,79^. Nevertheless, increased susceptibility to infections during a pandemic is still a threat. The slow viral clearance, induced lung damage, and increased mortality are all underlying mechanisms. Patel et al., concluded that corticosteroid drugs are useful in COVID-19, with a few limitations^80^.

Results of our study also showed a statistically significant negative correlation between the need for hemodialysis and the average CsA level (*p* value 0.019). A statistically significant negative correlation between the need for hospitalization and the average Tac level (*p* value 0.046). Some studies suggest that immunosuppressive drugs could be harmful in the early phase of COVID-19 when an immune response is essential to guard against viral replication. On the other hand, immunosuppressive drugs might be of use, during the “cytokine storm^62^”. Schapiro et al. showed that wait-listed patients with COVID-19 needed more hospitalization, and had higher mortality, compared to a transplant group, on immunosuppressive therapy^74^. The evidence from the SARS and MERS outbreak shows that the immunocompetent and the immunocompromised have similar outcomes, regarding morbidity and mortality from COVID-19^81^. The RECOVERY trial concluded that the use of long-term immunosuppressive drugs for solid organ transplants carries no increased risk of mechanical ventilation or mortality, except for Rituximab^24^. Moreover, some studies proved the anti-viral properties of many immunosuppressive drugs, like Cyclosporine and mycophenolate against SARS-CoV^82,83^.

If lung affection in COVID‐19 is caused partly by overactive T cells, therefore, impairment in the function of T cells as in immunosuppression might decrease lung injury^32^.

CsA and TAC, in vitro, form complexes with cyclophilins and other binding proteins, decreasing the activation of T cells^84^. Cyclophilins alter cellular and viral proteins^85^, essential for viral replication^83,84^. Importantly, this effect is dose-dependent^31,86–88^. Both decrease interleukin‐2 production, which is essential for the survival of T cells^89–91^. Romanelli and Mascolo concluded that immunosuppressive medications might be a “protective factor” for serious COVID-19^32^. However, studies to demonstrate these effects in vivo are still lacking^86,92–94^.

### Limitation

The main limitation of this study is the limited number of patients. Studies on a larger scale are needed to confirm the findings.

## Conclusion

In summary, this prospective study discussed the COVID-19 infection in Egyptian renal transplant recipients. Severity was related to baseline serum creatinine, serum LDH, Ferritin, AST, ALT and cumulative steroid dose. The study also highlighted that the immunocompromised state of renal transplant recipients may not be regarded as a disadvantage in the setting of COVID-19 infection. A concept with a lot of controversy during this pandemic. A concept that is of value not only in COVID19 pandemic, but also in the study of future serious viral infections in immunocompromised patients. Studies on a larger scale are needed to validate these results.
