# Author Correction: Outcome of COVID-19 in Egyptian living-donor kidney transplant recipients and relation to maintenance immunosuppressive drugs: a pilot study

**DOI:** 10.1038/s41598-023-48743-9

**Published:** 2023-12-19

**Authors:** Maggie Said ElNahid, Marianne Samir Makboul Issac, Khaled Marzouk Sadek

**Affiliations:** 1 Department of Internal Medicine and Nephrology, Faculty of Medicine, Cairo University, Cairo, Egypt; 2 Department of Clinical and Chemical Pathology, Faculty of Medicine, Cairo University, Cairo, Egypt

Correction to: *Scientific Reports* 10.1038/s41598-023-45750-8, published online 03 November 2023

The original version of this Article contained an error in Table 2, where the median (range) value was incorrect for ‘Cumulative steroid dose (mg)’ in the ‘Yes (n = 20)’ column. The correct and incorrect value appears below.

Incorrect:

**Table:** 

Patients’ data	Severity		*p* value
	No (n = 11)	Yes (n = 20)	
Cumulative steroid dose (mg)	5 (5–205)	5 (5–210)	0.001

Correct:

**Table:** 

Patients’ data	Severity		*p* value
	No (n = 11)	Yes (n = 20)	
Cumulative steroid dose (mg)	5 (5–205)	205 (5–210)	0.001

The original Article has been corrected.

